# Clinical and Histopathological Findings in a Patient of Darier–White Disease with Acrokertasosis Verruciformis of Hopf

**DOI:** 10.1155/2022/5233837

**Published:** 2022-07-04

**Authors:** Vikash Paudel, Manish Bhakta Pradhan, Brijesh Shrestha, Sumit Paudel

**Affiliations:** ^1^Department of Dermatology and Venereology, National Medical College, Birgunj, Nepal; ^2^Department of Pathology, National Medical College, Birgunj, Nepal

## Abstract

Darier disease (DD) is a rare genodermatoses characterized by greasy hyperkeratotic papules in seborrheic regions and nail and oral changes. Histologically, it presents as suprabasal clefts with acantholytic and dyskeratotic cells. Acrokertasosis verruciformis of Hopf (AKVH) is considered an allelic variant with clinical overlap where Church spires are seen histologically without dyskeratoses. Patients are susceptible to various viral and bacterial skin infections requiring prevention and treatment of infection. Nonspecific treatment includes patient counseling on exacerbating factors. Although there are no curative treatments for DD, topical corticosteroids and systemic retinoids may be used to control inflammation and hyperkeratosis. We are reporting a rare case with clinical and histological findings of DD with AKVH in a 17-year-old boy with keratotic papules, presented on the hands and feet, nose, and ears without mucosal involvement.

## 1. Introduction

Darier disease (DD) or Darier–White disease is an autosomal dominant genodermatosis, characterized by greasy hyperkeratotic papules in the seborrheic area and nail and mucous membrane changes [1]. First described by Prince Marrow (1886) and reported independently by Darier and White (1889), there is a mutation in the ATPA2 gene [2, 3]. Acrokeratosis verruciformis of Hopf (AKVH) is an autosomal dominant disorder of keratinization, described by Hopf (1931) [4] and is characterized by multiple flat-topped, papules on the hands and feet, punctate keratosis, and varying degrees of nail involvement [5]. DD and AKV are regarded as genetic evidence of unitary origin with allelic similarities, and it is suggested that patients with DD frequently show lesions that are indistinguishable from AKVH, both clinically and histopathologically [6–8]. We are reporting a rare case of DD with AKVH in a 17-year-old male who was treated with oral isotretinoin with a favorable outcome.

## 2. Case Report

**Figure 1:**
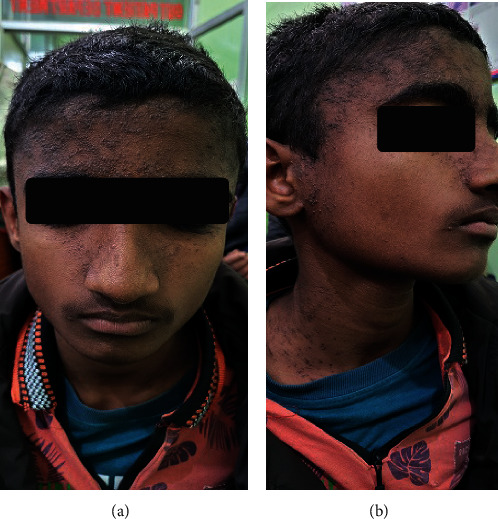
(a, b) Multiple flat-topped brownish verrucous plaques over the forehead, neck, and the periauricular area.

A 17-year-old adolescent male, presented with generalized pruritic eruptions for 3 months. Initially, the lesions started from the neck later to progress over the face and trunk. No family history of atopy or such illness was found. On examination, there were firm, greyish, warty and greasy papules, and plaques scattered over the forehead, scalp, postauricular and preauricular regions, external ear, neck, nasolabial folds, anterior chest, and scapular area (Figures 1(a) and 1(b)).

**Figure 2:**
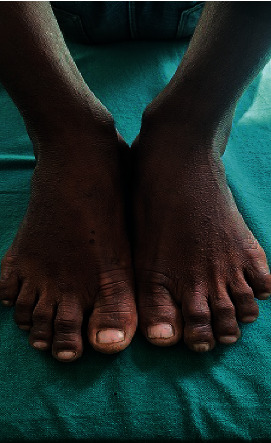
Multiple keratotic papules over the dorsum of bilateral feet.

**Figure 3:**
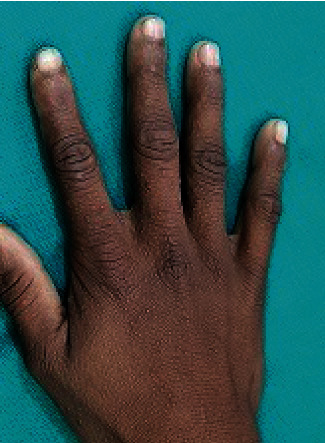
Multiple keratotic papules over the dorsum of the hand.

Small-sized warty papules were also noted over the dorsum of the bilateral hands and feet (Figures 2 and 3). Oral mucosa appeared to be normal.

Routine investigations were within normal limits. Skin punch biopsies were taken from the neck and dorsum of the left hand for histopathological examination. Clinical and laboratory findings were compatible with DD and AKVH, respectively. However, genetic analyses and functional analyses of the mutation could not be done because of economic constraints, thus allelic similarities between the two could not be established.

**Figure 4:**
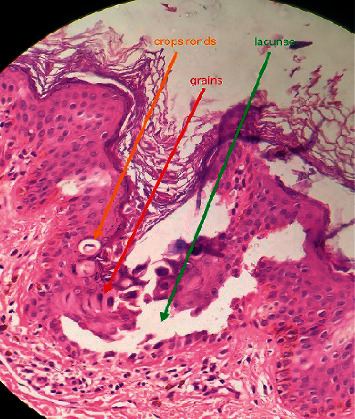
H&E stain with 20×: acantholysis and dyskeratosis represented by corps ronds and corps grains in DD.

**Figure 5:**
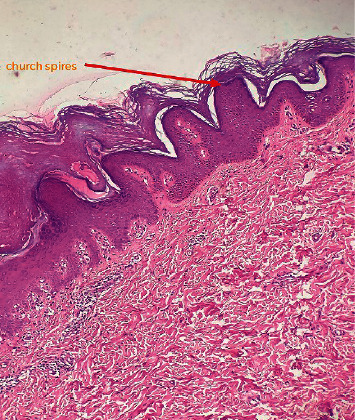
H&E stain with 10×: focal areas of hyperkeratosis, papillomatosis, and acanthosis with church spire appearance in AKVH.

Histopathological examination of the lesion from the neck shows acantholysis and dyskeratosis represented by corps rods and corps grains (Figure 4). Microscopic examination of the lesion from the dorsum of the left hand shows a focal area of hyperkeratosis, papillomatosis, and acanthosis with church spire appearance which was compatible with AKVH (Figure 5).

The patient was treated with isotretinoin 20 mg once daily, 5% salicylic acid and tretinoin 0.025% sunscreen, and urea-based moisturizers. Within two weeks of therapy, the lesions were flattened and crusted lesions were reduced. The patient was under follow-up for 3 months with improvement in the lesion, later the patient was lost.

## 3. Discussion

Darier's disease is a rare autosomal dominant genodermatosis caused by a mutation in the ATPA2 gene, at chromosome 12q23–12q24, which encodes the sarcoplasmic endoplasmic reticulum Ca^2+^ ATPase type 2 protein (SERCA2). The main function of SERCA2 is transporting Ca^2+^ from cytosol to the lumen of the endoplasmic reticulum [5]. A defect of SERCA2 leads to a deficiency of Ca^2+^ at the cell membrane, resulting in impaired cell-to-cell adhesion and apoptosis [5, 9]. The prevalence of this disorder in the population is 1 : 100,000. The sex incidence is equal, although the males appear to be more severely affected than females [10].

The clinical features of DD include the presence of multiple, hyperkeratotic papules distributed in seborrheic areas, as in our patient. The other clinical characteristics include flexural vegetative lesions, wart-like papules on the dorsal side of the hands and feet, palmer plantar pits, with red and white longitudinal bands, the distal wedge shape of the nail plate, and cobblestone papules on the oral mucosa. The patient might present with a foul offensive odor as well due to bacterial contamination. The oral mucosa is affected in 50% of the cases and lesions are usually asymptomatic [11]. Oral mucosal involvement and typical nail involvement were not present in our patient.

AKVH patients include mutations in ATP2A2, suggesting that this condition may be a variant of DD. However, it has been disputed for years that AKV and DD are separate entities or part of the same disease process. The main argument in favor of AKV and DD being separate entities is the existence, as in this family, of pure pedigrees where members only have features of AKV. In favor of AKVH being part of the DD spectrum is the presence of mixed pedigrees, and it is notable that 50% of Darier's patients have acral warty papules representing AKVH [7].

It is characterized by multiple flat-topped, flesh-colored papules on the hands and feet, punctate keratosis on the palms and soles, with varying degrees of nail involvement [7]. Our patient has similar lesions over the dorsum of the hand suggestive of AKVH.

Histopathological features of Darier's disease include acantholysis and dyskeratosis represented by “corps ronds” and “grains” (Figure 4). Corps ronds are located in the granular cell layer of the epidermis as central round dyskeratotic basophilic masses surrounded by a clear halo-like zone. Electron microscopy demonstrates loss of desmosomes, breakdown of keratin intermediate filament attachment, and perinuclear aggregation of keratin intermediate filaments [8]. Histopathological features of AKVH include papillomatosis with circumscribed epidermal elevations known as “church spire,” acanthosis, hyperkeratosis, and hypergranulosis without parakeratosis (Figure 5) [8]. The histopathological findings in our patient were compatible with AKVH as well.

The differential diagnosis includes seborrheic dermatitis, acne vulgaris, acanthosis nigricans, confluent reticulate papillomatosis, and prurigo pigmentosa. Histologically, the disease needs differentiation from Hailey Hailey disease, Grover's disease, and pemphigus vulgaris. Immunofluorescence of skin biopsy differentiates different acantholytic disorders [5].

Treatment of DD remains a challenge due to a lack of validated curative treatment guidelines. Several treatment modalities have been reported in the literature including systemic/topical retinoids, corticosteroid, cyclosporine, fluorouracil, derma-abrasion, electro surgery, ablative lasers, photodynamic therapy, and surgical excision; however, they provide limited effectiveness [12]. Systemic retinoids have proven better in achieving some reduction of symptoms in 90% of patients. They reduce hyperkeratosis, smoothen papules, and reduce odor. Thus, isotretinoin was used in our patient with a good outcome. Sunscreen and emollients are also crucial [12]. Therefore, it is important to ensure a multidisciplinary approach in the management of patients with Darier's disease.

Though our case is supported by its clinical picture and histologic finding, its main limitation is the lack of genetic, molecular and functional analyses to confirm and correlate the allelic similarities between DD and AKVH.

Regardless of the clinical severity and treatment option, the patient should receive genetic counseling with information on the inherited condition and risk of transmission to the offspring. A biopsy is fundamental to allow a final diagnosis based on this result. Patients should be informed of the complications of this disorder and the care required. These patients should be treated by a multidisciplinary team.

## Data Availability

Data not required for case reports.
